# Data-driven modeling and identification of a bistable soft-robot element based on dielectric elastomer

**DOI:** 10.3389/frobt.2025.1546945

**Published:** 2025-07-17

**Authors:** Abd Elkarim Masoud, Jürgen Maas

**Affiliations:** Mechatronic Systems Laboratory, Institute of Machine Design and Systems Technology, Technische Universität Berlin, Berlin, Germany

**Keywords:** bistable dielectric elastomer actuator, RBF network, dynamical and hybrid modeling, soft robot, smart structure

## Abstract

This paper presents the development and experimental validation of a hybrid modeling framework for a bistable soft robotic system driven by dielectric elastomer (DE) actuators. The proposed approach combines physics-based analytical modeling with data-driven radial basis function (RBF) networks to capture the nonlinear and dynamic behavior of the soft robots. The bistable DE system consists of a buckled beam structure and symmetric DE membranes to achieve rapid switching between two stable states. A physics-based model is first derived to describe the electromechanical coupling, energy functions, and dynamic behavior of the actuator. To address discrepancies between the analytical model and experimental data caused by geometric asymmetries and unmodeled effects, the model is augmented with RBF networks. The model is refined using experimental data and validated through analytical, numerical, and experimental investigation.

## 1 Introduction

The emerging field of soft robotics offers the potential to replace traditional articulated robots made of rigid joints and materials, such as electric motors and piezoceramic actuators, with soft actuators functioning as artificial muscles in robotic systems. Dielectric elastomer transducers (DETs) are a prominent class of soft actuators capable of deforming under applied voltage. These actuators mimic biological muscles, excelling in attributes such as large deformation (Li et al., 2013), high energy density (Duduta et al., 2019), and fast response times (Chen et al., 2019). DETs consist of a soft dielectric material sandwiched between two flexible electrodes, with layer thicknesses in the micrometer range. Functionally, DETs act as deformable capacitors, relying on electrostatic stress to achieve significant deformation upon sufficient voltage application (Pelrine et al., 2000).

DE-based actuators have been widely adopted in the development of soft robotic systems (Guo et al., 2021), including grasping mechanisms (Kofod et al., 2007; Wang et al., 2019), walking robots (Eckerle et al., 2001; Pelrine et al., 2002), flying robots (Lau et al., 2014), and humanoid robots (Kovacs et al., 2007).

Bistable soft robotic systems have attracted significant research attention due to their energy-efficient and versatile design potential, particularly in applications requiring rapid and reliable switching between two stable states. These actuators are well-suited for a variety of applications, including robotic systems, valve controls, and gripper mechanisms, where precise and consistent actuation is essential (Cao Y. et al., 2021; Chi et al., 2022; Harne and Wang, 2013). A range of bistable DE transducer designs have been proposed, highlighting their adaptability for diverse use cases. Examples include digital mechatronic systems with multiple degrees of freedom (Wingert et al., 2006), compact configurations combining cross-shaped bistable elements with conical DE actuators (Follador et al., 2015a), and bio-inspired soft grippers based on DE minimum energy structures Wang et al. (2019). Another design involves a bistable DE actuator comprising a buckled beam and two vertically arranged DE roll actuators to drive a rigid plate (Baltes et al., 2022). These designs are often supported by analytical and finite element models to study their static and dynamic behaviors. A common feature in many bistable DE designs is the use of buckled beams, valued for their mechanical bistability and reliability. Additionally, membrane geometries such as diamond, conical, roll, or strip shapes allow for tailored actuator performance to meet specific application requirements (Follador et al., 2015a; Follador et al., 2015b).

Beside, advancements in modeling and simulation of DETs have addressed their complex nonlinear behaviors. Physics-based approaches have been extensively utilized to describe the static behavior of DEs, considering hyperelastic material properties and electrostatic coupling (Suo, 2010; Zhang et al., 2006) as well as static hysteresis effects (Mertens et al., 2024). Finite element methods (FEM) have been used to capture the influence of mechanical boundary conditions, geometric inhomogeneities, and complex DE configurations (Wissler and Mazza, 2005; Kuhring et al., 2015). Furthermore, advanced dynamic continuum models have been introduced, accounting for nonlinear electromechanical coupling (Hansy-Staudigl et al., 2019). In addition to FEM-based methods, control-oriented modeling approaches have emerged, treating DEs as systems with lumped parameters. These models incorporate viscoelastic behaviors, represented by Kelvin-Voigt, Maxwell elements and address electrical losses from resistances (Sarban and Jones, 2012; Jones and Sarban, 2012; Hackl et al., 2005). Specific dynamic models have been proposed for various DE configurations, including stack actuators (Hoffstadt and Maas, 2015; Masoud et al., 2021), membrane actuators with spring preloading (Rizzello et al., 2016), and bistable robot modules featuring roll DE actuators (Soleti et al., 2023). For bistable DE systems, dynamic vibration and bifurcation analyses of circular membranes with springs were further investigated (Wang Z. et al., 2024; Cao C. et al., 2021).

Despite the widespread success of physics-based methods, there is a notable shift toward data-driven approaches for modeling soft robot systems, using advancements in machine learning techniques (Kim et al., 2021). Purely data-driven methods, such as radial basis function networks, recurrent neural networks (RNNs), and multilayer perceptrons, have been employed to model complex nonlinear behaviors without requiring explicit knowledge of underlying physical principles (Chen et al., 2024; Melingui et al., 2014). These approaches rely on empirical training, achieving high accuracy in capturing system dynamics.

Hybrid methods, combining data-driven machine learning with physics-based models, have also gained attention. These include frameworks that integrate machine learning components with existing first-principles models (Johnson et al., 2021) and physics-informed neural networks (PINNs), designed to embed physical laws directly into the learning process (Liu et al., 2024; Wang X. et al., 2024; Sun et al., 2022). Such approaches offer a balance between empirical accuracy and interpretability, enhancing the robustness of nonlinear system modeling. In the context of dielectric elastomer actuators (DEAs), neural network architectures such as long short-term memory (LSTM) networks (Xiao et al., 2020), radial basis function networks (Xiao et al., 2023), and gated recurrent units (GRUs) (Zhang et al., 2023) have been employed to model dynamic and nonlinear behaviors, including hysteresis and creep effects. Additionally, self-sensing models for DEAs have been developed using nonlinear autoregressive networks with exogenous inputs, enabling predictive modeling of displacement without requiring external sensors (Huang et al., 2023). While these data-driven approaches exhibit excellent alignment with experimental data, they often lack explicit incorporation of physical principles, underscoring the trade-off between predictive accuracy and interpretability.

This paper focuses on the development of a physics-based and data driven dynamic model for a bistable soft robot based on DE actuators. In this work, a similar structure to that in (Follador et al., 2015b) is investigated, but with a different constructive and kinematic design. While (Follador et al., 2015b) incorporated two lateral beams and two parallel DEs (consisting of VHB) in the center, our design features a single central beam with two laterally arranged DE membranes made of silicone. Similar structures and ideas have been previously introduced in (Masoud and Maas, 2023), where a variant is implemented in this paper for model validation and analyzed in detail. While previous studies employed a static model for designing the bistable DE actuator, this paper presents a systematic design approach that not only considers the static behavior but also incorporates dynamic effects, external forces, and a comprehensive description of the kinematics. The model is developed to be directly applicable to further system and control engineering applications. Moreover, the models investigated in this paper are directly compared with experimental data, with a particular focus on analyzing the transient response and accounting for inaccuracies in the entire actuator design. The derivation of the governing equations follows the framework presented in (Masoud and Maas, 2023), providing a foundation for analyzing the DE system’s dynamic behavior.

To enhance the accuracy and generalizability of the model, a hybrid approach is introduced that integrates physics-informed structures with data-driven methodologies. Specifically, the model is refined and augmented using artificial neural networks trained with experimental data, with a focus on radial basis function (RBF) networks. The RBF network is chosen for its ability to handle nonlinearities in a straightforward and interpretable manner, offering an effective means of capturing the complex behaviors of the bistable DE actuator.

The further contribution of this paper is structured as follows. Section 2 introduces the operating principles and design details of the bistable DE actuated system. Section 3 presents the physics-based modeling approach, analyzing the essential characteristics of the soft system and offers a reduced-order model under analytical considerations. In Section 4, a hybrid modeling approach is outlined, where the physics-based model is enhanced with a RBF neural network to effectively capture nonlinearities and uncertainties of the bistable DE systems. Section 5 provides a comprehensive validation of the proposed model through numerical simulations and experimental data. Finally, Section 6 summarizes the findings.

## 2 Concept and design of the bistable dielectric elastomer

**FIGURE 1:**
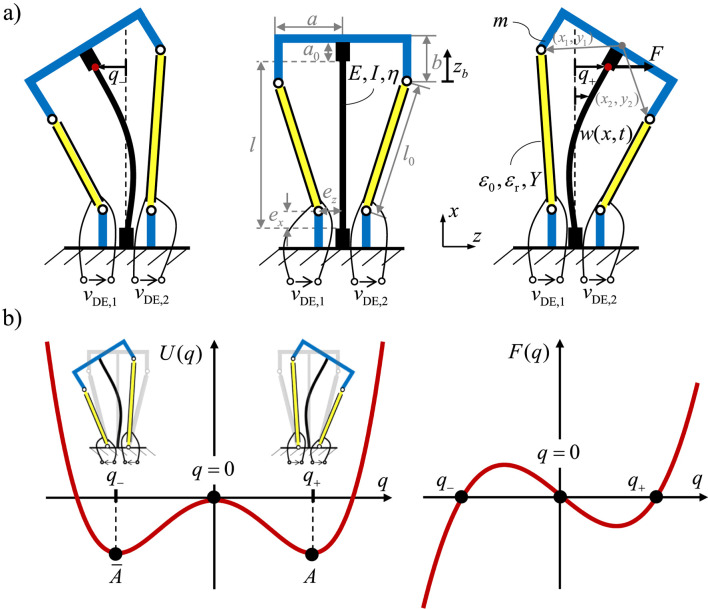
Concept of the bistable DE actuated system: **(a)** Schematic Representation with geometric dimensions and material parameters, **(b)** potential energy and force curves.

Figure 1 illustrates the concept and behavior of the proposed bistable DE-actuator, highlighting both its mechanical configuration and its bistable characteristics through potential energy and force curves. Figure 1a shows the actuator system in its three possible states. The system consists of a buckled beam, two symmetric DE membrane, and a moving mass (blue) that connects the elastic structure to the DEs. The DE membranes are pre-stretched such that the beam assumes one of two stable, buckled positions, either to the left or to the right. The application of a voltage ( $v_{\text{DE,}1}$ or $v_{\text{DE,}2}$ ) to one of the DE actuators generates a force that drives the system from one stable equilibrium state to the other. The central position (q=0) represents an unstable equilibrium point. The beam’s horizontal displacement q defines its position, where shifts $q_{-}$ and $q_{+}$ correspond to the two stable states. The DE membranes actively deform when voltage is applied, providing the necessary force to shift the black buckled beam between these configurations.

Figure 1b visualizes the bistable nature of the system through potential energy U(q) and force F(q) curves. The potential energy U(q) illustrates a double-well profile, with two minima at $q_{-}$ and $q_{+}$ , corresponding to the two stable equilibrium positions. The peak at q=0 indicates an unstable equilibrium state, acting as a threshold the system must overcome to transition between the two wells. The force curve F(q) , derived as the gradient of the potential energy curve $\frac{\partial U\left(q\right)}{\partial q}$ , further illustrates this bistable behavior. At $q_{-}$ and $q_{+}$ , the force is zero. At q=0 , the force changes direction, driving the system toward one of the two stable states, depending on the applied input.

**FIGURE 2:**
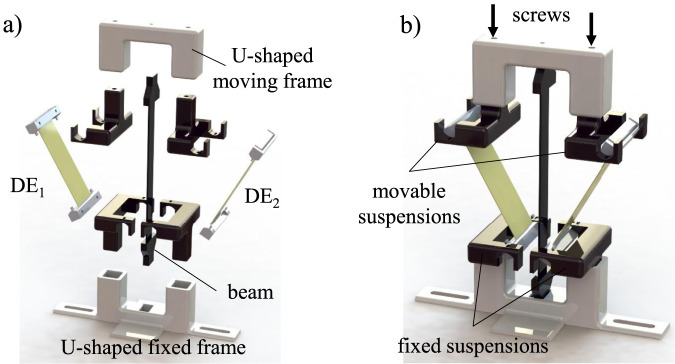
Design of the bistable DE actuated system: **(a)** exploded view and **(b)** assembly.

The CAD design of the bistable DE system is illustrated in Figure 2. The exploded view shows the arrangement of the system’s parts. The U-shaped lower fixed frame serves as a platform for mounting two black suspensions on its right and left sides, which are used to attach the two DE membranes at their lower ends. The DE actuators are designed in a strip-like configuration and arranged and arranged symmetrically. Centrally positioned within this frame is a slender beam made of PETG, which forms the buckled axis capable of toggling between two stable positions.

The actuator’s active elements are two symmetrical DE membranes and produced as multilayer laminates using the manufacturing process (Krüger et al., 2023). For the experimental investigations presented in this paper, we used the dielectric elastomer material ELASTOSIL® 2030 (EL 2030) (Wacker, 2016), which has a layer thickness of 50 μm and a dielectric breakdown strength ranging from 80 V/μm to 100 V/μm . As the electrode material, we employed the carbon black-filled elastomer ELASTOSIL® LR 3162 (EL 3162) (Wacker, 2022), which was applied using a jetting process (Cabuk and Maas, 2021). The effective breakdown strength typically decreases after fabrication processes such as electrode application, curing, and handling. To ensure long-term stability and high durability of the actuator, the maximum electric field is therefore conservatively limited to 60 V/μm . Given the film thickness of 50 μm , this corresponds to an applied voltage of 3 kV . This operational limit is well below the breakdown threshold of the processed material and has proven effective in practice for maintaining device reliability over multiple actuation cycles.

The two membranes, held in dedicated mounts, deform in response to applied voltage, thereby actuating the buckled beam. The two upper black mounts connect the two DE actuators at their upper ends, which are linked to a freely movable U-shaped frame. At this upper section of the actuator, two adjustment screws enable precise control of the pre-strains in the DE membranes and allow for fine-tuning of the membrane tension. The pre-stretched DEs are a critical feature, as it enables precise calibration of the actuator’s initial state and facilitates smooth transitions between its two stable states. Various connecting components, including screws and fasteners, ensure the precise alignment and structural integrity of the system.

The fully assembled soft DE system in Figure 2b presents a compact design tailored for precise functionality. The central buckled structure is symmetrically clamped by the DE membranes, which are configured to tilt the axis to either side based on the activated voltage. The design is particularly well-suited for demanding soft material applications that require consistent, adaptable joint performance and the flexibility to integrate with various structural configurations.

## 3 Modeling and analysis of bistable dielectric elastomer

In the following, the bistable DE actuator shown in Figure 1 is analyzed, and a dynamic model is derived. The figure visualizes the geometric dimensions and material parameters of the system. The modeling approach presented in (Masoud and Maas, 2023) is applied here. In the first step, the kinematic deformation of the DEs are introduced. Subsequently, the energy and virtual work terms for the DE actuators and the beam element are formulated, taking into account the mechanical and electrical behavior of the model. The dynamics of the electrical behavior are neglected in this paper. The Rayleigh-Ritz approximation is employed to describe the deformation of the beam, incorporating various boundary conditions and defining the generalized coordinates. Finally, the Hamiltonian principle is applied to the electromechanical system, resulting in a nonlinear system of differential equations. This system can be conveniently transformed into a state-space model for further analysis. Geometric nonlinearities are considered to accurately describe the unstable buckling behavior of the beam element, which leads to multiple equilibrium solutions. These solutions are a characteristic feature of the actuator concept and play a crucial role in its bistable performance.

### 3.1 Deformation of the DEs

To describe the deformation of the DE system in the deformed state, the geometric quantities depicted in Figure 1 are introduced. These quantities are used to calculate the kinematics of the actuators and the forces acting on them as a result of the deformation. The deformation of the DE actuator is described by the pitch angle φ , which captures the rotation of the system due to the change in the deflection w(x) in the x -direction. This pitch angle is given by:

$$\varphi =\arctan\left({\left.\frac{\partial w}{\partial x}\right|}_{l}\right),$$ (1)

where $\frac{\partial w}{\partial x}$ is the derivative of the deflection w(x) with respect to x at the position l , representing the deformation in the x -direction. The vertical displacement $u_{y}$ of the system is obtained from the initial displacement $u_{0}=\varepsilon_{0}l$ , with the displacement due to the rotation of the beam subtracted:

$$u_{y}=u_{0}-\int_{0}^{l}\frac{1}{2}{\frac{\partial w}{\partial x}}^{2}dx.$$ (2)

The displacement

$$q=w\left(x=l\right)$$ (3)

represents the deflection at the position x=l and is later selected as the generalized coordinate. The geometric positions of the left and right boundaries of the DE actuator, $x_{1},y_{1}$ and $x_{2},y_{2}$ , as shown in Figure 1, can be calculated as a function of the inclination angle φ :

$$\begin{array}{cccc} x_{1} & =a\cos\left(\varphi \right)+b\sin\left(\varphi \right), & y_{1} & =b\cos\left(\varphi \right)-a\sin\left(\varphi \right),\\ x_{2} & =a\cos\left(\varphi \right)-b\sin\left(\varphi \right), & y_{2} & =b\cos\left(\varphi \right)+a\sin\left(\varphi \right), \end{array}$$ (4)

where a and b are constants related to the geometry of the system as shown in Figure 1. The lengths of the DE actuators in the deformed state, $l_{\text{DE,}1}$ for the left actuator and $l_{\text{DE,}2}$ for the right actuator, are calculated using the above-defined geometric quantities. These lengths are derived as the Euclidean distances between the deformed positions:

$$\begin{array}{cc} l_{\text{DE,}1} & =\sqrt{{\left(-e_{z}-q-a_{0}\sin\left(\varphi \right)-x_{1}\right)}^{2}+{\left(e_{x}-l-u_{y}+a_{0}\cos\left(\varphi \right)-y_{1}\right)}^{2}},\\ l_{\text{DE,}2} & =\sqrt{{\left(e_{z}-q-a_{0}\sin\left(\varphi \right)+x_{2}\right)}^{2}+{\left(e_{x}-l-u_{y}+a_{0}\cos\left(\varphi \right)-y_{2}\right)}^{2}}, \end{array}$$ (5)

where $e_{x}$ , $e_{z}$ and $a_{0}$ are constants defining the geometry of the system. From the deformed lengths of the actuators, $l_{\text{DE,}1}$ and $l_{\text{DE,}2}$ , the stretches $\lambda_{1,1},\lambda_{2,1},\lambda_{3,1}$ for the left actuator and $\lambda_{1,2},\lambda_{2,2},\lambda_{3,2}$ for the right actuator can be calculated assuming an uniaxial deformation state:

$$\begin{array}{cccccc} \lambda_{1,1} & =\frac{l_{\text{DE,}1}}{l_{0}}, & \lambda_{2,1} & =\frac{1}{\sqrt{\lambda_{1,1}}}, & \lambda_{3,1} & =\lambda_{2,1},\\ \lambda_{1,2} & =\frac{l_{\text{DE,}2}}{l_{0}}, & \lambda_{2,2} & =\frac{1}{\sqrt{\lambda_{1,2}}}, & \lambda_{3,2} & =\lambda_{2,2}, \end{array}$$ (6)

where $\lambda_{1}$ represents the strain in the longitudinal direction of the respective DE actuator, while $\lambda_{2}$ and $\lambda_{3}$ correspond to the strains in the transverse and thickness directions, respectively. The kinematic relations presented in Equations 1–6 are used in the subsequent calculations of energy and work expressions.

### 3.2 Energy function and virtual work of the bistable DE system

To investigate the dynamic behavior of the bistable DE system, the Lagrangian function is derived:

$$L=T-\;U_{\text{s}}-\;U_{\text{m}}-\;U_{\text{DE,}1}-\;U_{\text{DE,}2}.$$ (7)

It includes the kinetic energy T , the strain energy of the beam $U_{\text{s}}$ , and the potential energy of the mass $U_{\text{m}}$ as well the two n-layer DEs $U_{\text{DE,}1}$ and $U_{\text{DE,}2}$ .

#### 3.2.1 Kinetic energy

The kinetic energy T of the system consists of the kinetic energy of the mechanical connection in the upper part of the system and the beam element B . The mechanical connection is modeled as a concentrated mass element m . The kinetic energy of the beam is simplified using the assumptions of the Euler-Bernoulli beam theory. The total kinetic energy of the system is the sum of the kinetic energies of the mass element and the beam:

$$T=\frac{1}{2}m\left({\dot{q}}^{2}+{\dot{u}}_{y}^{2}\right)+\frac{1}{2}\int_{0}^{l}\rho \left(I{\dot{w}}^{\prime^{2}}+A{\dot{w}}^{2}\right)dx.$$ (8)

Here, ρ is the mass density of the beam, A denotes the cross-sectional area of the beam, and $I=\int_{A}z^{2}dA$ is the area moment of inertia of the beam’s cross-section. The first term in the integral represents the kinetic energy associated with the rotation of the cross-sectional plane around the beam’s neutral axis. The second term describes the translational kinetic energy resulting from the displacement w(x,t) in the z -direction.

#### 3.2.2 Strain energy of the beam and potential energy of the mass

The strain energy of the beam represents the elastic energy stored in the material due to deformations. It is formulated as a function of the displacement w and its derivatives $w^{\prime }$ and $w^{\prime \prime }$ . The strain energy is expressed as:

$$U_{\text{s}}=\int_{0}^{l}EIw^{{\prime \prime }^{2}}\left(1+w^{\prime^{2}}\right)dx+\frac{1}{2}\frac{EA}{l}u_{0}^{2}.$$ (9)

Here, the first term represents the energy due to bending stress, while the second term corresponds to the contribution from axial strain. EI is the bending stiffness, and EA represents the axial stiffness of the cross-section. Additionally, the potential energy of the mass m is considered, given by:

$$U_{m}=m\cdot g\cdot u_{y}$$ (10)

where g represents the gravitational acceleration, and $u_{y}$ denotes the vertical displacement of the mass.

#### 3.2.3 Free energy of the DE actuators

The free energy of the two DE actuators $U_{\text{DE,}1}$ , $U_{\text{DE,}2}$ are derived based on the Neo-Hookean model while assuming a uniform charge distribution across the dielectric area $A_{0}$ . The free energy is expressed as:

$$\begin{array}{cc} U_{\text{DE,}1} & =V_{\text{DE}}\frac{Y}{6}\left(\lambda_{1,1}^{2}+\frac{1}{\lambda_{1,1}^{2}}-2\right)-\frac{1}{2}\frac{C_{0}}{\lambda_{3,1}^{2}}v_{\text{DE,}1}^{2}\\ U_{\text{DE,}2} & =V_{\text{DE}}\frac{Y}{6}\left(\lambda_{1,2}^{2}+\frac{1}{\lambda_{1,2}^{2}}-2\right)-\frac{1}{2}\frac{C_{0}}{\lambda_{3,2}^{2}}v_{\text{DE,}2}^{2},\;\text{with}\;C_{0}=n\varepsilon_{0}\varepsilon_{\text{r}}\frac{\beta A_{0}}{d_{0}}. \end{array}$$ (11)

Here, $V_{\text{DE}}$ represents the DE actuator volume, Y is the Young’s modulus, $C_{0}$ is the capacitance in the initial undeformed state, $\varepsilon_{0}$ and $\varepsilon_{\text{r}}$ are the vacuum permittivity and relative permittivity, n is the number of DE layers, β is the area ratio between the electrode area and the total area of DE, while $v_{\text{DE,}1}$ , $v_{\text{DE,}2}$ denotes the voltages and correspond to the left and right actuators, respectively.

#### 3.2.4 Mechanical virtual work

The mechanical virtual work $\delta W_{\text{m}}$ describes the energy dissipation within the system, accounting for beam damping and viscous losses in the DE material (Kelvin-Voigt-Maxwell model) as well as the generated work due to the external Force F . This can be expressed in terms of w(x,t) , $\varepsilon_{3,1}=\lambda_{3,1}-1$ , $\varepsilon_{3,2}=\lambda_{3,2}-1$ and the Maxwell strains $\varepsilon_{\text{E,l}}$ , $\varepsilon_{\text{E,r}}$ :

$$\begin{array}{cc} \delta W_{\text{m}} & =-\int_{0}^{l}2\eta I{\dot{w}}^{\prime \prime }\delta w^{\prime \prime }dx+F\delta q\\ & \;+V_{\text{DE}}\left[\eta_{\text{MW}}\left({\dot{\lambda }}_{3,1}-{\dot{\varepsilon }}_{\text{E,}1}\right)-Y_{\text{MW}}\varepsilon_{\text{E,}1}\right]\delta \varepsilon_{\text{E,}1}\\ & \;-V_{\text{DE}}\left[Y_{\text{MW}}\varepsilon_{\text{E,}1}+\eta_{\text{v}}{\dot{\varepsilon }}_{3,1}\right]\delta \lambda_{3,1}\\ & \;+V_{\text{DE}}\left[\eta_{\text{MW}}\left({\dot{\lambda }}_{3,2}-{\dot{\varepsilon }}_{\text{E,}2}\right)-Y_{\text{MW}}\varepsilon_{\text{E,}2}\right]\delta \varepsilon_{\text{E,}2}\\ & \;-V_{\text{DE}}\left[Y_{\text{MW}}\varepsilon_{\text{E,}2}+\eta_{\text{v}}{\dot{\varepsilon }}_{3,2}\right]\delta \lambda_{3,2}, \end{array}$$ (12)

where η , $\eta_{\text{MW}}$ and $\eta_{\text{v}}$ are the damping coefficients of the beam and DE material, while $Y_{\text{MW}}$ represents the elastic modulus of the considered Maxwell element (Masoud and Maas, 2023).

The introduced energy and work terms form the basis for modeling the bistable DE system. This framework enables the analysis of the system’s static and dynamic behavior as well as its electromechanical coupling. To describe the system, the displacement w(x,t) , the axial displacement $u_{0}$ , and the Maxwell strains $\varepsilon_{\text{E,}1}$ and $\varepsilon_{\text{E,}2}$ are defined. These generalized coordinates provide a comprehensive representation of the deformation state of the DE system.

However, since w(x,t) represents a spatially continuous field, the resulting partial differential equations (PDEs) include spatial and temporal dynamics along with boundary conditions. Such PDEs are typically complex and unsuitable to design control systems due to their high-dimensional nature. To reduce the complexity of the PDEs and enable systematic analysis and controller design, the Ritz method is employed. This approach approximates the spatial deformation w(x,t) using polynomial functions, effectively eliminating spatial dependency. The resulting simplified model significantly facilitates mathematical handling and allows for the application of classical control design methods, enabling targeted system investigations.

### 3.3 Analysis of the bistable DE system

The geometric and material parameters used for this investigation are provided in Table 1.

**TABLE 1:** Geometry and material parameters of the bistable DE soft robot joint under investigation for model-based analyses.

DET			Beam			Geometry		
parameter	unit	value	parameter	unit	value	parameter	unit	value
$\varepsilon_{0}$	As/Vm	$8,854\cdot 10^{-12}$	l	mm	90	a	mm	32
$\varepsilon_{\text{r}}$	1	2.8	A	$mm^{2}$	11	b	mm	23
n	1	8	I	$mm^{4}$	1.1	$b_{\text{max}}$	mm	30
$d_{0}$	μm	50	E	MPa	1.62	$e_{x}$	mm	7
$l_{0}$	mm	51	ρ	$g/cm^{3}$	1.2	$e_{z}$	mm	16
$A_{0}$	$mm^{2}$	1,326	η	MPa⋅s	3.64	m	g	40
Y	MPa	1.2	$a_{0}$	mm	11			
$Y_{\text{MW}}$	MPa	0.31						
$\eta_{\text{MW}}$	kPa⋅s	83.5						
$\eta_{\text{v}}$	kPa⋅s	0.3						

The material parameters of the DE actuators (Neo-Hookean model with Y=1.01 MPa and Kelvin-Voigt-Maxwell model with $\eta_{\text{v}}=0.3\;kPa$ , $\eta_{\text{MW}}=0.19\;kPa$ , $Y_{\text{MW}}=0.11\;MPa$ ) were initially adopted from Mertens et al. (2024). The mechanical properties of both the elastomer and electrode were thoroughly measured using standard procedures as outlined by Carpi et al. (2015). A more complex material model could provide a more detailed and precise representation of the hyperelastic behavior of the silicone material. However, the Neo-Hookean model has been shown to adequately capture the material response for strains up to 50% (Mertens et al., 2024; Hoffstadt et al., 2018). Since the DE actuators used in this study operate within this range, the chosen model appears to be a reasonable approximation. Introducing a more complex material model would increase the analytical complexity without necessarily leading to significant improvements in accuracy for the given operating conditions. The relative permittivity $\varepsilon_{\text{r}}$ was taken from data sheet (Wacker, 2016) and is also used by other researchers for this DE material. Since the DE actuator is composed of a composite material consisting of elastomer and electrode materials, the parameters of the DE material are slightly higher than the specified values, see (Mertens et al., 2024).

Regarding the beam material, the elastic modulus E=1.6…2.1 MPa and mass density $\rho =1.27\;g/cm^{3}$ were obtained from PETG (2023). These values refer to the raw material of the filament. For 3D-printed objects made of PETG, the effective elasticity and density can vary due to printing parameters such as infill density, layer height, and potential air inclusions. As no data on damping properties for PETG was available we assumed a linear viscous damping with parameter η for the beam, see Equation 12.

Therefore, the parameters Y , $\eta_{\text{v}}$ , $\eta_{\text{MW}}$ , $Y_{\text{MW}}$ , E , ρ and η , were subsequently adjusted for experimental comparison using the complete actuator setup through an optimization process. The specified parameters served as initial values and the deviation between the model and the experiment was minimized using the sum of squared errors with a gradient-based method in MATLAB. The experimental data from Section 5 were used for parameter identification. It should be noted that the damping parameter η=3.64 MPa is significantly larger than the parameters $\eta_{\text{v}}=0.3\;kPa$ and $\eta_{\text{MW}}=83.5\;kPa$ . This indicates that the 3D-printed beam material exhibits stronger damping behavior, which dominates the viscous properties of the DE material. As will be shown in Section 5.2, the DE material does not determine the system behavior, but is dominated by the flexible beam structure, so that further model improvements in the DE domain have only a minimal influence on the overall behavior.

The static behavior of the bistable DE system is analyzed by focusing on the total potential energy of the DE system, dependent on deformation w(x,t) , the applied voltages $v_{\text{DE,}1}$ , $v_{\text{DE,}2}$ , and the external force F . In this analysis, kinetic energy and dissipative work terms are neglected to simplify the model. This approach allows for the characterization of the bistable DE system’s unique properties.

#### 3.3.1 Polynomial ritz approximation

To calculate the static deformation of the DE beam actuator, a fourth-order polynomial Ritz approximation is employed:

$$w\left(x\right)\approx w_{4}\left(x\right)=q_{2}x^{2}+q_{3}x^{3}+q_{4}x^{4}.$$ (13)

This approximation satisfies the boundary conditions and is chosen based on a balance between accuracy and model simplicity. Lower-order polynomials (<4) yield insufficient accuracy, while higher-order polynomials provide negligible improvements but increase computational complexity. Thus, the fourth-order approximation is a suitable compromise.

#### 3.3.2 Energy formulation using the Hamilton principle

The Hamilton principle is applied to the static electromechanical DE beam structure. The total potential energy of the system is expressed as:

$$\delta U=\delta U_{\text{s}}+\delta U_{\text{DE,}1}+\delta U_{\text{DE,}2}+\delta U_{\text{m}}=F\delta q,$$ (14)

considering Equations 9–11. substituting the Ritz approximation (Equation 13) into the total energy (Equation 14) and applying the principle of stationary energy, the equilibrium equations with respect to the generalized coordinates $q_{\text{m}}={\left[\begin{array}{cccc} q_{2} & q_{3} & q_{4} & u_{0} \end{array}\right]}^{T}$ are derived as:

$$\frac{\partial U}{\partial q_{\text{m}}}=\frac{\partial U_{\text{s}}}{\partial q_{\text{m}}}+\frac{\partial U_{\text{DE,}1}}{\partial q_{\text{m}}}+\frac{\partial U_{\text{DE,}2}}{\partial q_{\text{m}}}+\frac{\partial U_{\text{m}}}{\partial q_{\text{m}}}=F\frac{\partial q}{\partial q_{\text{m}}}.$$ (15)

#### 3.3.3 Influence of pre-strain on actuation behavior

The influence of pre-strain on the actuation behavior is analyzed by adjusting the geometric parameter b , see Figure 1. By varying b , the system is tuned to achieve an actuated bistable state. All other geometric and material parameters remain constant. The system parameters used for this investigation are provided in Table 1. The equilibrium positions for unactuated and actuated states are computed using the equilibrium equations across the range b∈[20 mm,30 mm] , with applied voltages of $\left(v_{\text{DE,l}}=v_{\text{DE,r}}=0\right)$ , $\left(v_{\text{DE,l}}=3\;kV,\;v_{\text{DE,r}}=0\;kV\right)$ and $\left(v_{\text{DE,l}}=0\;kV,\;v_{\text{DE,r}}=3\;kV\right)$ . These equations are solved for $q_{2}$ , $q_{3}$ , $q_{4}$ and $u_{0}$ using the Newton method in MATLAB.

**FIGURE 3:**
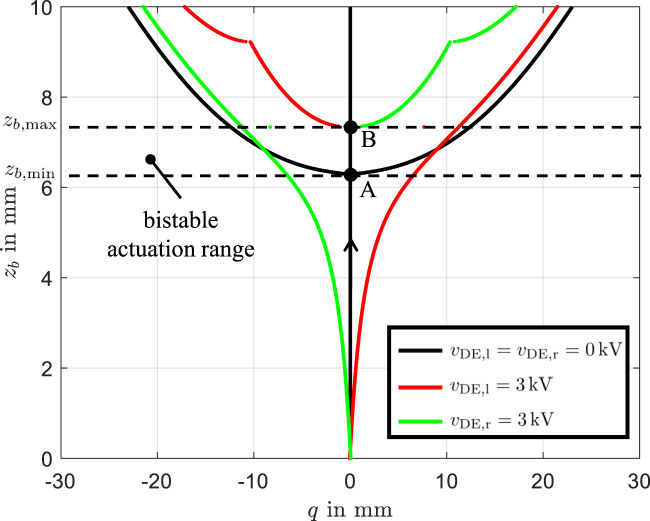
Equilibrium states of the DE-actuator system as a function of the position $z_{b}$ for the voltages $v_{\text{DE,l}}=v_{\text{DE,r}}=0kV$ (black lines), $v_{\text{DE,l}}=3kV,\;v_{\text{DE,r}}=0kV$ (red lines), and $v_{\text{DE,l}}=0kV,\;v_{\text{DE,r}}=3kV$ (green lines).

The results are presented in Figure 3, where the x-axis represents the maximum displacement:

$$q=q\left(q_{\text{m}}\right)=w\left(l\right)=q_{2}l^{2}+q_{3}l^{3}+q_{4}l^{4},$$ (16)

and the y-axis denotes the displacement parameter $z_{b}$ :

$$z_{b}=b_{\text{max}}-b.$$ (17)

For $z_{b}\leq z_{b,min}$ , the beam is stable in its vertical position until a critical point A . Beyond this point $\left(z_{b}>z_{b,min}\right)$ , the vertical equilibrium becomes unstable, and small disturbances cause the beam to snap to either a left-leaning or right-leaning stable configuration. This bifurcation creates three equilibrium paths: two stable operating points and one unstable point at q=0 .

When a voltage of $v_{\text{DE,}1}=3\;kV$ is applied to the left DE actuator, the equilibrium states shift horizontally from the black curve to the red curve. Similarly, activating the right DE actuator with $v_{\text{DE,}2}=3\;kV$ shifts the equilibrium states to the green curve. As seen in Figure 3, the region between $z_{b,min}$ and $z_{b,max}$ exhibits bistability, allowing the beam to switch between equilibrium states depending on the applied voltage. For $z_{b}>z_{b,max}$ , bistability persists, but the beam cannot perform transition between stable states via electrical actuation of DEs. The practical operating range for the bistable DE system lies between $z_{b,min}$ und $z_{b,max}$ , as shown in Figure 3. Within this range, electrical excitation can effectively toggle the system between stable states. Beyond this range, the DE system remains locked in one stable state, limiting its practical functionality.

#### 3.3.4 Force-displacement characteristic

**FIGURE 4:**
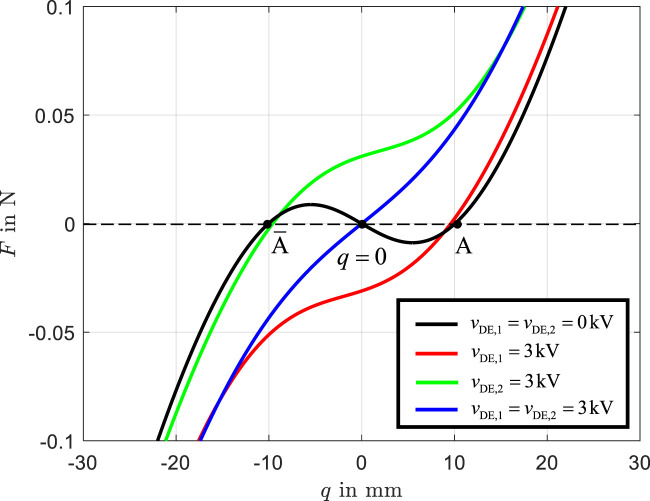
Force-displacement characteristic F(q) for the voltages $v_{\text{DE,l}}=v_{\text{DE,r}}=0kV$ (black line), $v_{\text{DE,l}}=3kV,\;v_{\text{DE,r}}=0kV$ (red line), $v_{\text{DE,l}}=0kV,\;v_{\text{DE,r}}=3kV$ (green line), and $v_{\text{DE,l}}=v_{\text{DE,r}}=3kV$ (blue line).

The force-displacement relationship of the bistable beam actuator in the direction of q , is analyzed as shown in Figure 4. To facilitate this analysis, the force F is varied along the q -axis.

Using Equation 15, the system is solved for $q_{2}$ , $q_{3}$ , $q_{4}$ and $u_{0}$ over a range of forces $F\in \left[-100\;\text{mN},100\;\text{mN}\right]$ . The displacement q is then determined. As shown in Figure 4, the force-displacement curve is represented for the following voltage states:

• $v_{\text{DE,l}}=v_{\text{DE,r}}=0\;kV$ (black curve),

• $v_{\text{DE,l}}=3\;kV,\;v_{\text{DE,r}}=0\;kV$ (red curve),

• $v_{\text{DE,l}}=0\;kV,\;v_{\text{DE,r}}=3\;kV$ (green curve), and

• $v_{\text{DE,l}}=v_{\text{DE,r}}=3\;kV$ (blue curve).

The black curve illustrates the unactuated state, where the actuator exhibits two stable equilibrium positions at A and $\bar{A}$ , with an unstable equilibrium at q=0 . When the left actuator is activated, the beam transitions to the right stable state (A) . Similarly, activating the right actuator causes the beam perform transition to the left stable state $\left(\bar{A}\right)$ . If the actuator is deactivated, the system remains at the previous stable state. When both actuators are simultaneously activated, the unstable equilibrium at q=0 becomes stable. This results in the blue force-displacement curve, highlighting the significant effect of simultaneous actuation on the stability and equilibrium characteristics of the system.

#### 3.3.5 Evaluation of the capacitance-displacement relationship

**FIGURE 5:**
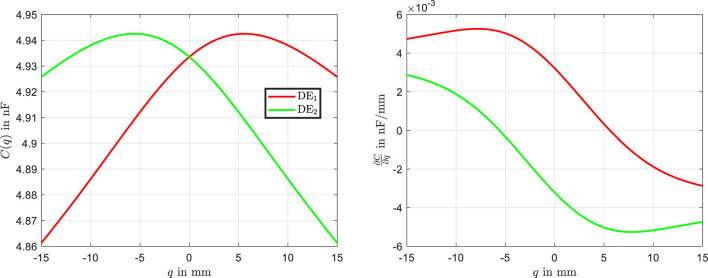
Capacitance-displacement characteristics of the left and right DE actuators, along with their derivatives with respect to q .

The capacitance-displacement characteristics, $C_{1}\left(q_{\text{m}}\right)$ for the left actuator and $C_{2}\left(q_{\text{m}}\right)$ for the right actuator, are analyzed as functions of the beam’s displacement q . The results are illustrated in Figure 5, which also includes the derivatives of the capacitance with respect to q . These derivatives play a critical role in determining the electromechanical forces generated by the DE actuators.

The electromechanical force $F_{\text{e}}$ acting on the beam in the q -direction is calculated using the capacitance $C_{1}$ and $C_{2}$ . This relationship is expressed as:

$$F_{\text{e}}=\frac{\partial U_{\text{DE,}1}^{\text{e}}}{\partial q}+\frac{\partial U_{\text{DE,}2}^{\text{e}}}{\partial q}=\frac{1}{2}\frac{\partial C_{1}}{\partial q}v_{\text{DE,}1}^{2}+\frac{1}{2}\frac{\partial C_{2}}{\partial q}v_{\text{DE,}2}^{2},$$ (18)

where $U_{\text{DE,}1}^{\text{e}}$ and $U_{\text{DE,}2}^{\text{e}}$ represent the stored electric energy in the DEs. As shown in Equation 18, the applied voltages $v_{\text{DE,}1}$ and $v_{\text{DE,}2}$ influences the force quadratically, while the direction of the force depends on the sign of $\frac{\partial C_{1}}{\partial q}$ and $\frac{\partial C_{2}}{\partial q}$ .

From Figure 5, it is evident that the derivative of the capacitance for the left actuator is predominantly positive. This results in a net force in the positive q -direction, causing the actuator to move toward the stable equilibrium on the right side. Conversely, the derivative of the capacitance for the right actuator is predominantly negative, generating a force in the negative q -direction. As a result, the actuator moves toward the stable equilibrium on the left side. These observations align with the bistable nature of the system, where the actuators forces shift the beam between the two equilibrium states based on the applied voltages.

The characteristic curves offer an intuitive way to understand the physical behavior of the bistable DE actuator. They serve as a basis for explaining the system’s force generation and equilibrium transitions. Additionally, these insights are critical for comprehending the actuator’s dynamic behavior, which will be explored in the following section. By analyzing these characteristics, simplifications can be introduced to model the dynamic behavior more effectively, ensuring a better understanding of the actuator’s performance under varying operating conditions.

### 3.4 Data-based reduced order state space model

The dynamic behavior of the bistable DE actuator can be described analogously to the approach in (Masoud and Maas, 2023) neglecting the electrical equations. The governing equation is expressed as:

$$\begin{array}{cc} & M{\ddot{q}}_{\text{m}}+D{\dot{q}}_{\text{m}}+f_{\text{F}}\left(q_{\text{m}}\right)+\bar{K}q_{\varepsilon }=\frac{1}{2}\frac{\partial C_{1}}{\partial q_{\text{m}}}v_{\text{DE,}1}^{2}+\frac{1}{2}\frac{\partial C_{2}}{\partial q_{\text{m}}}v_{\text{DE,}2}^{2}+F\frac{\partial q}{\partial q_{\text{m}}},\\ & \;{\dot{q}}_{\varepsilon }=K^{T}{\dot{q}}_{\text{m}}-Y_{\text{MW}}\eta_{\text{MW}}^{-1}q_{\varepsilon }, \end{array}$$ (19)

considering Equations 7–12. Using the state variables:

$$\begin{array}{ccccc} x & =\left[\begin{array}{c} q_{\text{m}}\\ {\dot{q}}_{\text{m}}\\ q_{\varepsilon } \end{array}\right], & u=v_{\text{DE}}, & & y=g\left(q_{\text{m}}\right) \end{array}$$ (20)

the system can be transformed into the state-space form:

$$\begin{array}{cc} \dot{x} & =f\left(x,u\right),\\ y & =g\left(x,u\right), \end{array}$$ (21)

where

$$\begin{array}{cc} f\left(x,u\right) & =\left[\begin{array}{c} {\dot{q}}_{\text{m}}\\ M^{-1}\left(\tau \left(q_{\text{m}},v_{\text{DE}},F\right)-D{\dot{q}}_{\text{m}}-f_{\text{F}}\left(q_{\text{m}}\right)-\bar{K}q_{\varepsilon }\right)\\ K^{T}{\dot{q}}_{\text{m}}-Y_{\text{MW}}\eta_{\text{MW}}^{-1}q_{\varepsilon } \end{array}\right],\\ g\left(x,u\right) & =q=\left[\begin{array}{cccc} l^{2} & l^{3} & l^{4} & 0 \end{array}\right]q_{\text{m}} \end{array}$$ (22)

and

$$\tau \left(q_{\text{m}},v_{\text{DE}},F\right)=\frac{1}{2}\frac{\partial C_{1}}{\partial q_{\text{m}}}v_{\text{DE,}1}^{2}+\frac{1}{2}\frac{\partial C_{2}}{\partial q_{\text{m}}}v_{\text{DE,}2}^{2}+F\frac{\partial q}{\partial q_{\text{m}}}.$$ (23)

Equation 21 accounts for the specific parameters of the DE system, represented by the mass matrix $M\left(q_{\text{m}}\right)\in R^{4\times 4}$ , the damping matrix $D\left(q_{\text{m}}\right)\in R^{4\times 4}$ , the restoring force $f\left(q_{\text{m}}\right)$ , and the creep matrix $\bar{K}\left(q_{\text{m}}\right)=V_{\text{DE}}Y_{\text{MW}}K\left(q_{\text{m}}\right)\in R^{4\times 2}$ .

When the focus is on motion in a measurable direction q , and it is known that only the first mode is significantly excited while others can be neglected, the state-space model can be reduced to this primary coordinate without altering the system’s essential characteristics. Through the static analyses, it has been observed that the horizontal motion q predominantly governs the system’s behavior, motivating a reduction to this observable coordinate.

**FIGURE 6:**
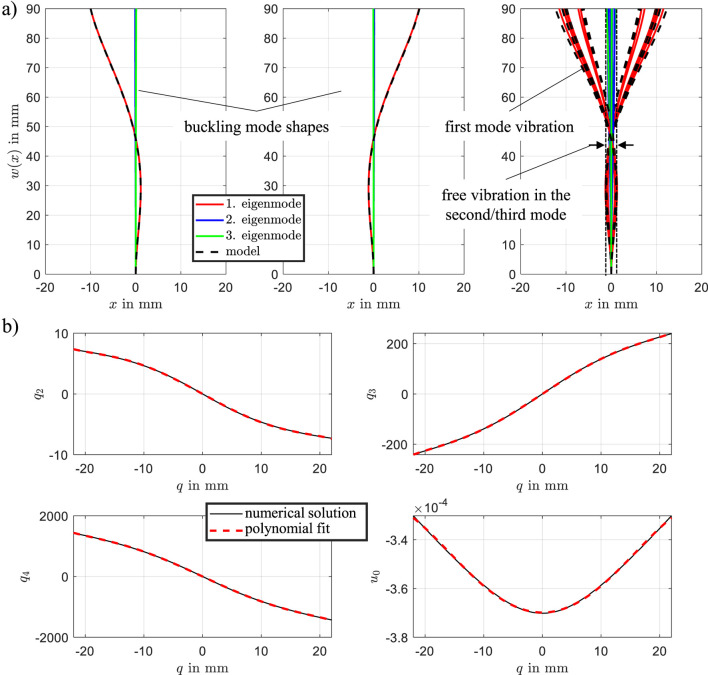
**(a)** Identified mode shapes of the beam during vibration. **(b)** State vector $q_{\text{m}}$ as a function of q . The polynomial fit captures the dependency for analytical representation.

**FIGURE 7:**
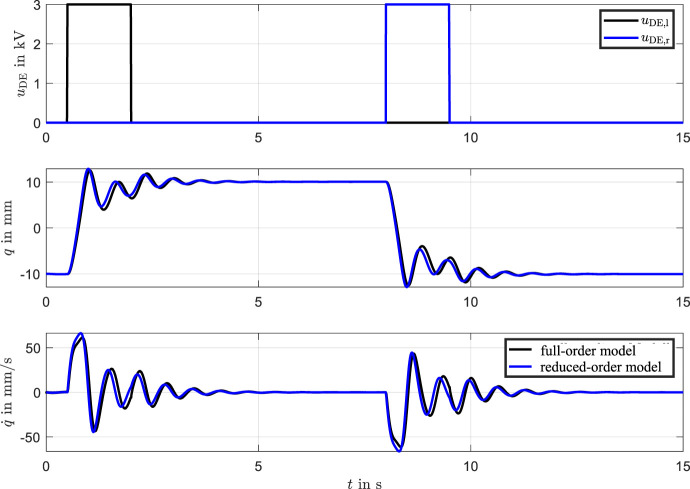
Comparison of simulations between the full-order and reduced-order models. The alignment confirms the reduced model’s accuracy in capturing key dynamics.

To analyze the system’s eigenmodes, simulation data from the model defined in Equation 21 were evaluated using singular value decomposition (SVD). The system was excited using the input signals shown in Figure 7, where the left actuator was activated first, followed by the right. The resulting beam dynamics were simulated accordingly. Figure 6a presents the identified mode shapes. The model response (black dashed line) closely matches the first mode shape (red line), while the second and third modes are nearly negligible. The right figure illustrates the mode shapes during transient oscillation. Here, the second and third modes exhibit significantly lower amplitudes compared to the dominant first mode, indicating that the transient dynamics are primarily governed by the first eigenmode. A slight deviation between the model and the first mode is observed under dynamic conditions. Overall, the analysis demonstrates that the bistable DE actuator’s behavior can be accurately captured by the first eigenmode alone, which supports the validity of reducing the model to a single generalized coordinate q .

The reduction of the system model is achieved by using the equilibrium solutions of the static model. The state vector $q_{\text{m}}$ is expressed as a function of the generalized coordinate q , as illustrated in Figure 6b. The mathematical formulation is given by:

$$q_{\text{m}}\left(q\right)=\left[\begin{array}{cccc} q_{2}\left(q\right) & q_{3}\left(q\right) & q_{4}\left(q\right) & u_{0}\left(q\right) \end{array}\right].$$ (24)

The static solutions depicted in Figure 6b are approximated using polynomial functions. This fitting enables the derivation of an analytical expression for $q_{\text{m}}\left(q\right)$ , facilitating its integration into the Ritz approximation.

By substituting Equation 24 into the approximation

$$w\left(x,t\right)=q_{2}\left(q\right)x^{2}+q_{3}\left(q\right)x^{3}+q_{4}\left(q\right)x^{4},$$ (25)

the Hamilton principle is then applied based on the measurable generalized coordinate q , utilizing the steps outlined in previous sections. The dynamic equations are reformulated as:

$$\begin{array}{cc} & m_{q}\ddot{q}+d\left(q\right)\dot{q}+f\left(q\right)+\left({\bar{k}}_{1}\left(q\right)q_{\varepsilon ,1}+{\bar{k}}_{2}\left(q\right)q_{\varepsilon ,2}\right)=\frac{1}{2}\frac{\partial C_{1}}{\partial q}v_{\text{DE},1}^{2}+\frac{1}{2}\frac{\partial C_{2}}{\partial q}v_{\text{DE},2}^{2},\\ & {\dot{q}}_{\varepsilon ,1}=k_{1}\left(q\right)\dot{q}-\frac{Y_{\text{MW}}}{\eta_{\text{MW}}}q_{\varepsilon ,1},\;{\dot{q}}_{\varepsilon ,2}=k_{2}\left(q\right)\dot{q}-\frac{Y_{\text{MW}}}{\eta_{\text{MW}}}q_{\varepsilon ,2}. \end{array}$$ (26)

$m_{q}$ represents the total mass, including the contribution of the beam’s partial mass. The term d(q) denotes a nonlinear damping characteristic, while f(q) corresponds to the restoring force. Additionally, ${\bar{k}}_{1}=V_{\text{DE}}Y_{\text{MW}}k_{1}$ and ${\bar{k}}_{2}=V_{\text{DE}}Y_{\text{MW}}k_{2}$ describe the creep behavior of the DEs. From this simplified Equation 26, the characteristic curves can be visualized effectively. Simulation results, comparing the high-dimensional and reduced-order models, are shown in Figure 7. The close agreement between the curves demonstrates that the reduced-order model effectively captures the dominant dynamics of the bistable DE actuator. This not only validates the reduction approach but also highlights its ability to significantly reduce computational complexity. This reduction approach facilitates efficient system analysis and control design, allowing the system’s behavior to be described through its most influential generalized coordinate q .

## 4 Data-driven modeling using RBF-Networks

Once the bistable DE has been designed and is available for further control, the mathematical model can alternatively be identified through experimental data aimed at accurately representing real-world behavior. The model presented in the previous section was used to design the actuator and perform analytical investigations and simulations to gain a better understanding of its behavior. However, in real-world implementations, deviations from the model can arise due to several factors. These include undetermined geometric asymmetries in the setup, such as imperfectly symmetric bearings, and unmodeled friction effects, such as hysteresis and dynamic friction, which are difficult to predict. These discrepancies necessitate significant post-experimental effort to adjust the model parameters, such as accounting for asymmetries in the geometry and fine-tuning the viscous parameters, in order to match the actual measurement data as closely as possible.

To address these challenges with comparably little effort, the model will be refined using experimental data and enhanced with RBF neural networks. This will allow for a more accurate representation of the actuator’s real-world performance. A generalized dynamic friction model will be incorporated to better account for the nonlinear and time-dependent frictional forces. Specifically, the viscous losses composed of the beam and DE previously described in the equations will be replaced with a generalized Maxwell-slip model (Al-Bender et al., 2005). This simple adjustment will improve the model’s ability to capture the complex friction dynamics and enhance its predictive accuracy.

The Hamiltonian principle is applied to the measurable coordinates q , $v_{\text{DE,}1}$ and $v_{\text{DE,}2}$ , formulating it with unknown energy terms. These unknown energy functions depend on both q and $\dot{q}$ , as well as on the measurable voltages $v_{\text{DE,}1}$ and $v_{\text{DE,}2}$ . However, the explicit functional relationships among these variables are not known and are modeled using RBF networks. The derivation of the corresponding equations is based on the Lagrange principle. The Lagrangian function is defined as follows:

$$\begin{array}{ll} & L\left(q,\dot{q},v_{\text{DE,}1},v_{\text{DE,}2}\right)=T\left(q,\dot{q}\right)\\ & \;-\left(U_{\text{mech}}\left(q\right)-U_{\text{DE,}1}^{\text{e}}\left(q,v_{\text{DE,}1}\right)-U_{\text{DE,}2}^{\text{e}}\left(q,v_{\text{DE,}2}\right)\right), \end{array}$$ (27)

where the kinetic energy T is given by:

$$\begin{array}{c} T=\frac{1}{2}m_{q}{\dot{q}}^{2}. \end{array}$$ (28)

The elastic energy of the beam and the hyperelastic behavior of the DEs are collectively represented by the potential energy of a nonlinear spring element

$$\begin{array}{c} U_{\text{mech}}=\int_{0}^{q}c\left(\tilde{q}\right)\tilde{q}\text{d}\tilde{q} \end{array}$$ (29)

This potential energy function encompasses both the mechanical strain energy of the beam and the nonlinear hyperelastic properties characteristic of DE materials, effectively unifying them within a single expression. The electrical energy stored in the left and right DE actuator can be represented using the energy expression for a deformable capacitor. In this approach, each DE actuator is modeled as a capacitor whose capacitance changes with deformation. This variable capacitance effectively captures the energy dynamics as the actuator undergoes shape changes. The electrical co-energy for each DE actuator $U_{\text{DE,}1}^{\text{e}}$ und $U_{\text{DE,}2}^{\text{e}}$ are given as follows:

$$\begin{array}{ccc} U_{\text{DE,}1}^{\text{e}} & =\frac{1}{2}C_{\text{DE,}1}\left(q\right)v_{\text{DE,}1}^{2},\;U_{\text{DE,}2}^{\text{e}} & =\frac{1}{2}C_{\text{DE,}2}\left(q\right)v_{\text{DE,}2}^{2}. \end{array}$$ (30)

For the derivation of the dynamic equations, co-energy is utilized since the voltages are directly measurable. To account for additional hysteresis effects and dynamic friction forces, observed in experimental laboratory tests, the model is expanded in accordance with (Al-Bender et al., 2005). Thus a generalized dynamic friction model is introduced, and an internal state equation is considered:

$$\begin{array}{cc} F_{\text{f}} & =F\left(q,\dot{q},z\right),\\ \dot{z} & =Z\left(q,\dot{q},z\right). \end{array}$$ (31)

Following the principles of the Lagrange formalism, the differential equations can be derived:

$$\begin{array}{cc} & \frac{\text{d}}{\text{d}t}\frac{\partial L\left(q,\dot{q},v_{\text{DE,}1},v_{\text{DE,}2}\right)}{\partial \dot{q}}-\frac{\partial L\left(q,\dot{q},v_{\text{DE,}1},v_{\text{DE,}2}\right)}{\partial q}=-F_{\text{f}}\left(q,\dot{q},z\right),\\ & \dot{z}=Z\left(q,\dot{q},z\right). \end{array}$$ (32)

This leads to the following equation:

$$\begin{array}{cc} & m_{q}\ddot{q}+F_{\text{f}}\left(q,\dot{q},z\right)+c\left(q\right)q=g_{\text{DE,l}}\left(q\right)v_{\text{DE,}1}^{2}+g_{\text{DE,}2}\left(q\right)v_{\text{DE,}2}^{2},\\ & \;\dot{z}=Z\left(q,\dot{q},z\right),\\ & \text{with}\\ & g_{\text{DE,}1}\left(q\right)=\frac{1}{2}\frac{\partial C_{\text{DE,}1}}{\partial q},\;g_{\text{DE,}2}\left(q\right)=\frac{1}{2}\frac{\partial C_{\text{DE,}2}}{\partial q}. \end{array}$$ (33)

Using the model in Equation 33, the nonlinear system can be experimentally identified by utilizing the measured quantities q , $\dot{q}$ , $\ddot{q}$ , $v_{\text{DE,l}}$ and $v_{\text{DE,r}}$ as well neural networks for the unknown functions. $\dot{q}$ and $\ddot{q}$ need to be determined through time derivative, which are often noisy. To derive the time derivatives from noisy signals, the Total Variational Differentiation (TVRegDiff) algorithm is utilized in machine learning frameworks to obtain smoother time derivatives Chartrand (2011).

### 4.1 Radial basis function network

**FIGURE 8:**
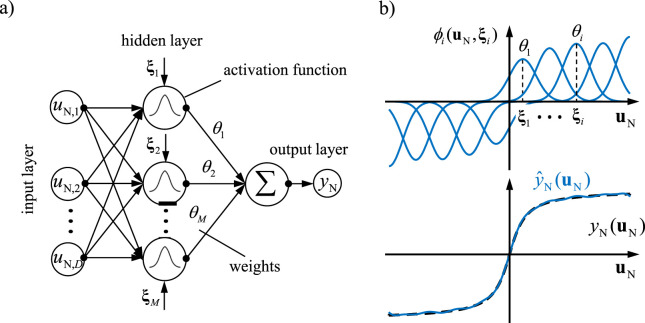
Radial basis function (RBF) network: **(a)** structure of the RBF network, **(b)** example of function approximation.

One approach to identify the nonlinearities in Equation 33 is to use radial basis functions, as illustrated in Figure 8a.

The RBF network consists of a hidden layer that contains neurons. The activation function of each neuron (radial basis functions $\phi_{i}$ ) is computed from its input values $u_{\text{N}}$ . The connections between neurons are scaled using weights $\theta_{i}$ , i=1,…,M , and the output is calculated through a weighted summation. The weights are the variable parameters of the neural network, determined through a learning process (e.g., least squares method, gradient method). A key feature of this network is the use of local basis functions $\phi \left(u_{\text{N}}\right)$ , shown at the top of Figure 8b. These radial basis functions exhibit the following expression:

$$\phi_{i}\left(u_{\text{N}}\right)=\phi \left(u_{\text{N}},\xi_{i}\right)=\phi \left(\left\|u_{\text{N}}-\xi_{i}\right\|\right),$$ (34)

where $\xi_{i}$ represents the radial centers, and $\left\|u_{\text{N}}-\xi_{i}\right\|$ denotes the Euclidean distance between $u_{\text{N}}$ and $\xi_{i}$ . The activation functions used here are based on Gaussian curves:

$$\phi_{i}\left(u_{\text{N}}\right)=e^{\left(\frac{E_{i}}{2\sigma^{2}}\right)},\text{ with}\;E_{i}=\|u_{\text{N}}-\xi_{i}{\|}^{2}={\left(u_{\text{N}}-\xi_{i}\right)}^{T}\left(u_{\text{N}}-\xi_{i}\right).$$ (35)

The variance σ is referred to as the smoothing factor, which determines the degree of overlap between neighboring activation functions $\varphi_{i}$ . Thus, a nonlinear function can be approximated using the RBF network as follows:

$$y_{\text{N}}\left(u_{\text{n}}\right)=\overset{M}{\underset{i=1}{\sum }}\theta_{i}\phi_{i}\left(u_{\text{n}}\right).$$ (36)

The basis functions transforms the D -dimensional input vector into a high-dimensional (M) feature space using Gaussian radial function. The output is simply a weighted sum of the basis functions (hidden layer).

An important feature of the RBF network is that it is nonlinear with respect to the input variables but linear with respect to the parameters. This property is especially advantageous for online parameter identification, as the linear dependency on the parameters allows for efficient real-time computation and adjustment. This means that the weights, which represent the network’s variable parameters, can be optimized in real-time using techniques such as the least squares method or gradient methods without requiring an extensive, computationally intensive optimization of the entire model structure.

This separation of nonlinearity in the input variables from linearity in the parameters also facilitates the stability and convergence of model adaptation. In practice, this property leads to improved adaptability of the RBF network, making it especially suitable for applications that demand dynamic and rapid adjustment to changing input data, such as in nonlinear dynamical models and nonlinear system control.

The radial basis function (RBF) network was selected due to its simple and interpretable structure, as well as the advantageous property that its parameters enter the model linearly. This linearity allows the identification problem to be formulated as a convex optimization task with a unique solution. In the presented approach, the RBF network is embedded within a physics-based model framework and serves to approximate nonlinearities arising from uncertainties or simplifications in the physical description. Since the dominant system dynamics are already represented by the physics-based component, the RBF network acts purely as a corrective term and does not need to reconstruct the full dynamics from data.

Alternative neural network architectures such as Long Short-Term Memory (LSTM) or Gated Recurrent Units (GRU) are more suitable for black-box modeling scenarios, where system dynamics are learned implicitly through internal memory mechanisms and time-delayed inputs. These models are inherently more complex, demand significantly more training data, and involve nonlinear optimization processes that are sensitive to initialization and may result in multiple local minima. Moreover, the integration of such recurrent structures into a physics-informed framework would necessitate a discrete-time formulation of the governing equations, which complicates both model development and training. In contrast, the use of RBF networks provides a transparent and efficient means of enhancing model accuracy while maintaining a strong connection to the underlying physical principles.

### 4.2 Dynamic identification of bistable DE system using physics-informed RBF network

The physical model should be as grounded in fundamental physical laws as possible while still utilizing neural networks to generalize any unknown functions effectively. This approach reduces the demand for extensive training data, striking a balance between the incorporation of physics-based structure and the amount of data required for neural network training. Achieving this compromise allows for accurate model identification with limited data, optimizing both computational efficiency and model accuracy.

Two approaches can be employed for identifying the nonlinear model in Equation 33. The first approach involves measuring characteristic curves with external integrated sensors and using neural networks to identify the unknown dynamic friction. Thus, a force sensor can be used to determine the force component c(q)⋅q in Equation 29. Position-dependent capacitance measurements $C_{\text{DE,}1}\left(q\right)$ and $C_{\text{DE,}2}\left(q\right)$ allow for the determination of characteristic curves $g_{\text{DE,}1}\left(q\right)$ and $g_{\text{DE,}2}\left(q\right)$ through differentiation. The inertial force can also be estimated, if the mass $m_{q}$ is known. By rearranging Equation 33 to solve for $F_{\text{f}}$ , the dynamic friction $F_{\text{f}}\left(q,\dot{q},z\right)$ can be identified, for example, using a Multi-Layer Perceptron (MLP) network (Demuth et al., 2014; Popescu et al., 2009). However, this approach requires significant hardware and measurement resources to accurately capture the characteristic curves. Moreover, parameterizing the neural network is complex, as it depends on multiple input variables and requires comprehensive excitation across the entire input space.

The second proposed approach involves predefining the structure of dynamic friction and representing all unknown functions using simple neural networks that are linear in the unknown parameters. To simplify the structure of dynamic friction, it is suggested in (De Wit et al., 1995; Dankowicz, 1999) to represent the state equation $Z\left(q,\dot{q},z\right)$ by a first-order low-pass filter to account for the delay of static friction force relative to the input velocity. Additionally, viscous damping is assumed by $F_{\text{f}}=dz$ , where $\dot{q}$ is replaced by the internal variable z :

$$\begin{array}{cc} m_{q}\ddot{q}+d\left(q\right)z+c\left(q\right)q & =\frac{1}{2}g_{\text{DE,}1}\left(q\right)v_{\text{DE,}1}^{2}+\frac{1}{2}g_{\text{DE,}2}\left(q\right)v_{\text{DE,}2}^{2},\\ \dot{z} & =\frac{1}{T_{z}}\left(\dot{q}-z\right). \end{array}$$ (37)

**FIGURE 9:**
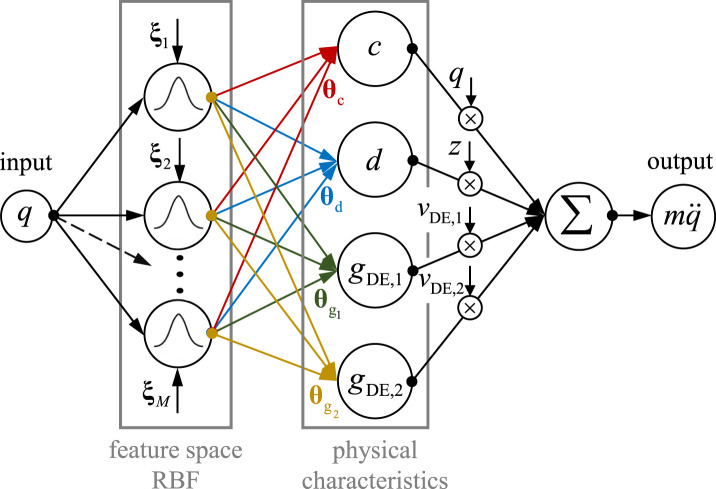
Physics-informed RBF network comprising the dynamics of the bistable DE transducer.

The nonlinear functions c(q) , d(q) , $g_{\text{DE,}1}\left(q\right)$ and $g_{\text{DE,}2}\left(q\right)$ can be identified using radial basis functions according to Equation 36 in Section 4.1:

$$\begin{array}{cc} c\left(q\right) & =\overset{M}{\underset{\text{i}=1}{\sum }}\phi_{i}\left(q\right){\hat{\theta }}_{\text{c},i},\\ d\left(q\right) & =\overset{M}{\underset{\text{i}=1}{\sum }}\phi_{i}\left(q\right){\hat{\theta }}_{\text{d},i},\\ g_{\text{DE,}1}\left(q\right) & =\overset{M}{\underset{i=1}{\sum }}\phi_{i}\left(q\right){\hat{\theta }}_{g_{1},i},\\ g_{\text{DE,}2}\left(q\right) & =\overset{M}{\underset{i=1}{\sum }}\phi_{i}\left(q\right){\hat{\theta }}_{g_{2},i},\\ \phi_{i}\left(q\right) & =\exp\left(-\frac{{\left(q-\xi_{i}\right)}^{2}}{2\sigma_{\text{n}}^{2}\Delta \xi^{2}}\right),\\ \Phi^{T}\left(q\right) & =\left[\begin{array}{cccc} \phi_{1}\left(q\right) & \phi_{2}\left(q\right) & \ldots & \phi_{M}\left(q\right) \end{array}\right]. \end{array}$$ (38)

where $\sigma_{\text{n}}$ is a the normalized smothing factor $\left(\sigma =\sigma_{\text{n}}\Delta \xi \right)$ . The radial centers $\xi_{\text{i}}$ are defined equidistantly according to the following equation:

$$\begin{array}{c} \xi_{\text{i}}=q_{\text{min}}+\left(i-1\right)\underset{\Delta \xi }{\underbrace{\frac{q_{\text{max}}-q_{\text{min}}}{M-1}}}, \end{array}$$ (39)

where $q_{\text{max}}-q_{\text{min}}$ is the operating Range. The complete physics-based RBF network for modeling and identifying the dynamic properties of the bistable DE system is depicted in Figure 9. This figure details the network’s structure, illustrating the integration of physical laws with radial basis functions (RBF) to capture nonlinear system dynamics.

The incorporation of fundamental physical principles allows the model to accurately represent key system characteristics, reducing the need for large data sets to train the neural network while enhancing generalizability. The RBF functions provide a flexible approach for modeling unknown nonlinear relationships within the system without extensive data requirements. This results in an efficient model structure that combines physics-based insights with data-driven optimization to enable reliable predictions of the DE dynamic responses.

Additionally, the figure illustrates the parameterization of individual components, including the weighting parameters $\theta_{\text{c}},\theta_{\text{d}}$ , $\theta_{g_{1}}$ and $\theta_{g_{2}}$ . These parameters, identified based on experimental data, represent the nonlinear characteristics of the system’s mechanical and electric components. Thus, Equation 37 can be transformed into the following form:

$$\begin{array}{cc} m_{q}\ddot{q} & =\left[\begin{array}{cccc} -\Phi^{T}\left(q\right)q & -\Phi^{T}\left(q\right)z & \Phi^{T}\left(q\right)v_{\text{DE,}1}^{2} & \Phi^{T}\left(q\right)v_{\text{DE,}2}^{2} \end{array}\right]\left[\begin{array}{c} {\hat{\theta }}_{c}\\ {\hat{\theta }}_{d}\\ {\hat{\theta }}_{g_{\text{DE,}1}}\\ {\hat{\theta }}_{g_{\text{DE,}2}} \end{array}\right],\text{ with}\\ m_{q}\ddot{q} & =\psi^{T}\hat{\theta },\;\ddot{q}\in R,\;\psi^{T}\in R^{1,4M},\;\hat{\theta }\in R^{4M,1}. \end{array}$$ (40)

The identification problem of the nonlinear system can be transformed into a linear regression problem using the RBF network, as the unknown parameters to be identified enter linearly, with the nonlinearities contained within the measurement vector. Here, the inertial force $y=m_{q}\ddot{q}$ is defined as the output variable.

The model parameters $\hat{\theta }$ in Equation 40 were estimated using a least squares (LS) approach, taking advantage of the model’s linearity with respect to these parameters. The time constant $T_{z}$ , which characterizes the system’s dynamic friction behavior, was identified through an iterative optimization procedure. In each iteration, the LS method was used to update $\hat{\theta }$ , while $T_{z}$ was refined via a gradient descent scheme until the norm of the modeling error converged. This sequential optimization approach enabled an effective calibration of the damping dynamics. The estimation problem is linear in $\hat{\theta }$ , resulting in a quadratic cost function that guarantees a unique solution. The parameter vector $\hat{\theta }$ was computed using the pseudoinverse of the regressor matrix. For the optimization of $T_{z}$ , convergence was evaluated by monitoring whether the cost function decreased below a predefined threshold. To ensure parameter identifiability and avoid numerical issues, the regressor matrix was verified to have full rank throughout the optimization, which was confirmed numerically by rank analysis.

## 5 Experimental validation

### 5.1 Experimental setup for identification and validation

**FIGURE 10:**
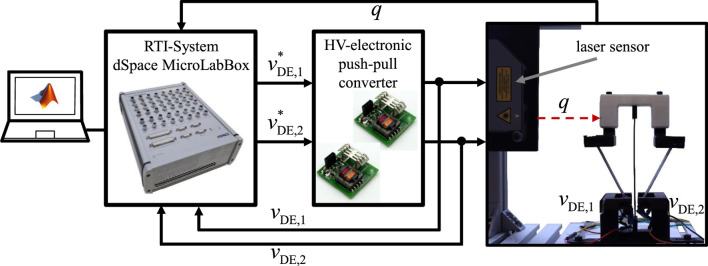
Experimental setup and hardware-in-the-loop system for the identification and validation of the bistable DE system.

The experimental setup for the characterization and validation of the bistable DE system is illustrated in Figure 10. The setup comprises the physical assembly of the bistable DE actuator, the electronic drive circuitry, a laser sensor, and the dSPACE real-time platform.

The pre-tension applied via screws serves a dual purpose: it must exceed the critical buckling load of the beam to maintain structural stability, yet it should not surpass a threshold beyond which the DE actuators cannot move the system between its two stable states. For the experiments, an operating point of b=23mm was selected, ensuring both bistability and actuation capability. The operational principle results in a rotational motion of the upper U-shaped profile, but only the horizontal component, q , is used as the measurement variable.

The horizontal displacement q is measured using a laser sensor (Model ILD2300-20) from Micro-Epsilon. This sensor has a measurement range of ±25 mm , a resolution of 0.3 μ m, a linearity deviation of 4  μ m, and a sampling frequency of 30 kHz . The sensor is visible to the left of the actuator in Figure 10.

The electrical actuation system employs two high-voltage (HV) drive circuits configured as push-pull converters, as described in (Junglas et al., 2022). These converters are HV DC-DC systems specifically designed for capacitive loads in the range of tens of nanofarads. Each converter can deliver a maximum voltage of 3 kV and operates with a 24 V DC input. With a peak output power of approximately 15 W , the system can rapidly charge and discharge the capacitive actuator. The control inputs are the reference voltages $v_{\text{DE,}1}*$ and $v_{\text{DE,}2}*$ , while the actual measured voltages, $v_{\text{DE,}1}$ and $v_{\text{DE,}2}$ , are available as outputs.

The programs are implemented in MATLAB and Simulink and compiled for execution on a MicroLabBox from dSPACE. This platform interfaces with both the drive electronics and sensors while providing a real-time data acquisition interface. It also allows on-the-fly modification of parameters such as setpoints and filter constants.

**FIGURE 11:**
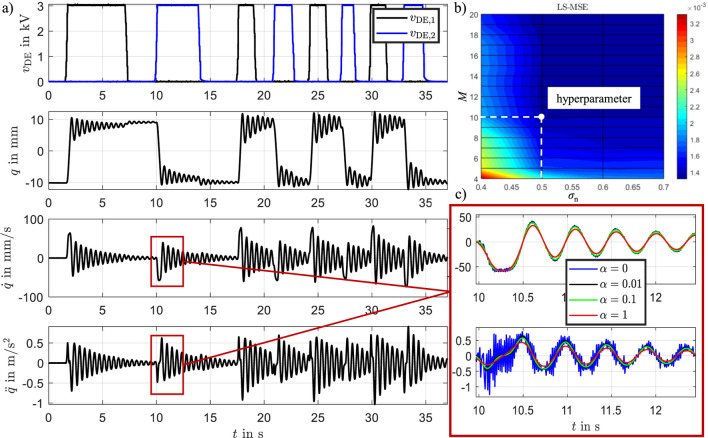
**(a)** Measurement data: position, velocity, and acceleration profiles, along with the measured excitation voltages applied to the bistable DE system, **(b)** sensitivity study of the mean squared error (MSE) with respect to the hyperparameters M and $\sigma_{\text{n}}$ , **(c)** TVRegDiff for different regularization parameter α .

Figure 11a displays the position, velocity, and acceleration profiles, along with the measured excitation voltages $v_{\text{DE}}$ applied to both actuators. The excitation profile for training the RBF network was designed to cover a broad frequency spectrum, ensuring comprehensive system excitation. It is essential to stimulate the entire measurement range adequately so that data is available for every position covered by the radial centers $\xi_{i}$ . This ensures that sufficient information is obtained across all radial centers, which is critical for accurate and effective system identification. The RBF model was identified using input-output data sampled at 10 ms intervals, yielding a total of 3750 data points. This was sufficient to identify the parameters of the hybrid model due to the incorporation of physical knowledge. A purely black-box approach (e.g., LSTM, GRU) would require significantly more experiments under varying operating conditions to ensure generalization, as well as extensive validation through multiple test scenarios.

To calculate the time derivatives of the measured signals in a robust manner, we applied the total variation regularized differentiation algorithm (TVRegDiff). The regularization parameter was set to α=0.01 , and a small constant $\varepsilon =10^{-6}$ was introduced to prevent division by zero during iterative updates. To assess the sensitivity of the method to the choice of α , we performed a parameter study as shown in Figure 11c. The results show that small values of α produce derivatives with high-frequency noise, while large values overly smooth the signal, leading to attenuation of important dynamic features. The red curve in Figure 11c illustrates this effect for α=1 . This analysis highlights the trade-off inherent in selecting the regularization parameter and justifies the chosen value, which balances noise suppression with temporal resolution.

The bistable behavior of the DE system is evident in the measurements. Starting from an initial position near q=−10 mm , an excitation voltage of $v_{\text{DE,}1}=3\;\text{kV}$ is applied to the left actuator. This action shifts the upper U-profile to the right stable position, which is maintained even after the voltage is removed, due to the system’s inherent bistability. The covered displacement range of ≈±12 mm highlights the large operational domain and the pronounced nonlinear behavior of the actuators within these bounds.

### 5.2 Results

The generated measurement data from Figure 11 were used to determine the parameters $\theta_{\text{c}},\theta_{\text{d}}$ , $\theta_{g_{1}}$ and $\theta_{g_{2}}$ according to Equation 40. For this purpose, M=10 radial basis functions were selected, and $\sigma_{\text{n}}=0.5$ was set. Each physical characteristic curve requires 10 parameters, resulting in a total of 40 parameters being identified using the least squares method. To analyze the influence of the RBF network hyperparameters on model performance, the number of basis functions M and the normalized smothing factor $\sigma_{\text{n}}$ were varied, and the resulting model accuracy was evaluated using the mean squared error (MSE). The results are presented in Figure 11b. The analysis shows that for M=10 and $\sigma_{\text{n}}=0.5$ , the model achieves a well-suited balance between accuracy and complexity. Increasing M or $\sigma_{\text{n}}$ beyond this point does not significantly improve the prediction quality but leads to greater overlap between basis functions, causing parameter redundancy and reduced identifiability.

**FIGURE 12:**
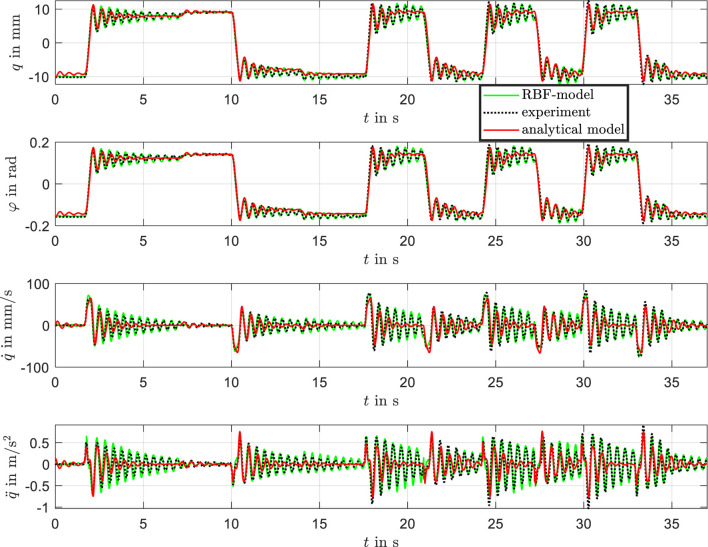
Comparison of measured data, analytical model, and RBF model of the bistable DE system.

The results are presented in Figure 12, comparing the measured position with the analytical model and the RBF-based model. The comparison includes position, velocity, and acceleration as well as the pitch angel φ . The time evolution of φ closely resembles that of the generalized coordinate q , given their functional relationship via the arctangent in Equation 1. For small angles, φ is approximately linearly proportional to q , which is clearly reflected in the experimental and simulated results shown in Figure 12.

For the following evaluation of the overall modeling, the statistical error measure normalized root mean squared error (NRMSE) was selected:

$$NRMSE\left(y\right)=\frac{\sqrt{\frac{1}{N}\sum_{k=1}^{N}{\left(y_{\text{meas},k}-y_{\text{calc},k}\right)}^{2}}}{y_{\text{max}}-y_{\text{min}}}.$$ (41)

Here, N denotes the number of samples, while $y_{\text{meas}}$ and $y_{\text{calc}}$ represent the measured and calculated signals, respectively. The comparison includes the analytical model and the physics-informed RBF network. The results are summarized in Table 2.

**TABLE 2:** NRMSE of the analytical model, RBF-model against experimental Data.

Model	NRMSE (q)	NRMSE (φ)	NRMSE $\left(\dot{q}\right)$	NRMSE $\left(\ddot{q}\right)$
analytical	5.7%	5.6%	10.4%	10.8%
RBF	2.1%	2.0%	4.1%	5.0%

The RBF-based model achieves a significantly lower NRMSE across all tested scenarios, particularly in the transient regime and during oscillatory behavior. This confirms that augmenting the analytical model with a data-driven RBF correction improves the model’s capability to capture nonlinearities and dynamic effects that are not fully represented in the analytical formulation.

**FIGURE 13:**
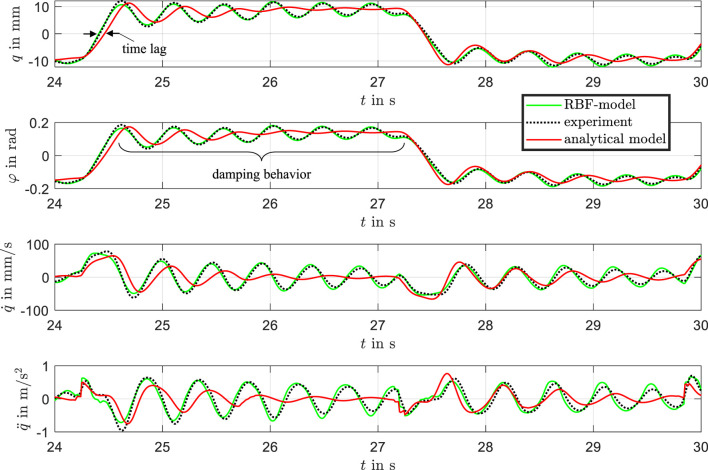
Zoomed-in comparison of measured data, analytical model, and RBF model in the time window from 24 to 30 s, highlighting differences in transient behavior.

A zoomed-in view of the data for the time interval between 24 and 30 s is shown in Figure 13, highlighting the dynamic differences between the models. Overall, the trained RBF network demonstrates excellent agreement with the experimental results.

While the analytical model captures the experimental data reasonably well and reproduces the static behavior effectively, it struggles to accurately represent the transient behavior during the settling process. This limitation stems from imprecise parameterization of the viscous effects and the presence of plastic effects in the beam material. For the analytical model, new parameters would need to be defined and optimized through a labor-intensive process for each actuator to improve accuracy. The analytical model exhibits a noticeable time lag and a stronger damping effect, which leads to an underestimation of overshoot and a failure to reproduce the oscillatory characteristics observed in the experimental data during fast input transitions. In contrast, the physics-informed RBF model closely matches the measured response, accurately capturing overshoot, phase delay, and the amplitude of oscillations. This improved performance demonstrates the capability of the RBF-model to represent complex nonlinear and transient dynamics that are not sufficiently captured by the analytical model. These findings support the hybrid modeling approach as an effective method for improving predictive accuracy in highly dynamic regimes.

**FIGURE 14:**
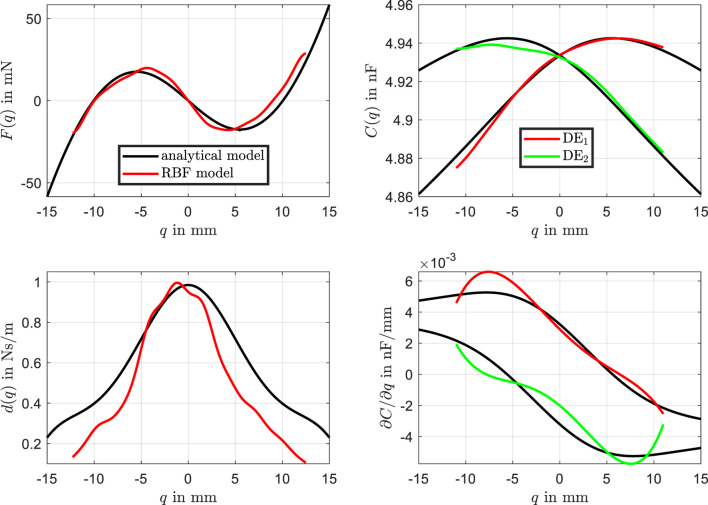
Physical characteristic curves. Comparison between the analytical curves (black lines) and the RBF-based curves (red and green lines for $DE_{1}$ and $DE_{2}$ , respectively).

Interestingly, the identification process using the RBF network allows for the direct derivation of the physical characteristic curves, as shown in Figure 14. The analytical characteristic curves (black lines) are compared with the RBF-based curves (red and green lines).

The RBF network captures the force characteristic curve with high accuracy, showing minimal deviation from the analytical model. The RBF-based curves for the two dielectric elastomer layers also align closely with the analytical model, demonstrating good agreement across the entire range. The most significant discrepancy is observed in the damping characteristic curve. This is where the model uncertainties, particularly related to dynamic and viscous effects of the beam, are most pronounced. This is due to the fact that the beam was modeled using a linear damping behavior, which is insufficient to improve modeling quality. One potential approach to enhance accuracy would be to explore alternative dynamic friction models for the beam and integrate them into the analytical model, which would likely yield more accurate results. However, this would involve a substantial modeling effort and would still not address other sources of inaccuracies and uncertainties.

Small asymmetries in the force and capacitance curves are evident, which are effectively captured by the RBF model but are absent in the analytical model. The comparison of the derived characteristic curves also includes the derivative of the capacitance $\frac{\partial C}{\partial q}$ , which exhibits some deviations between the analytical and RBF-based models. These discrepancies can be reasonably explained and are attributed to minor geometric deviations in the actuator assembly. The actuators are not perfectly identical, and the alignment of the bearings on the left and right sides of the actuator is not precisely matched. The RBF-based model successfully captures these variations, reflecting the influence of small asymmetries and non-idealities in the real system. In contrast, the analytical model assumes perfect symmetry and alignment, leading to a smoother representation that does not fully account for these subtle discrepancies.

The obtained results highlights the ability of the RBF network to follow the experimental data precisely, including dynamic transitions. These underscore the flexibility and accuracy of the RBF network, particularly in capturing nonlinearities and asymmetries that are not accounted for in the analytical model.

**FIGURE 15:**
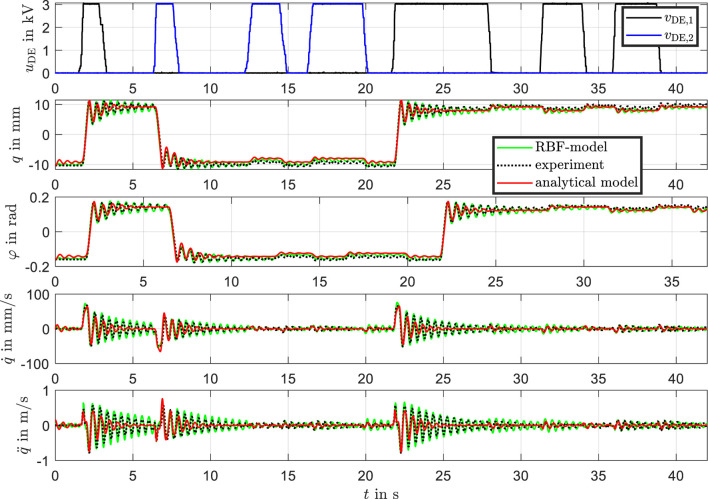
Cross-validation of measurement data, analytical model, and RBF model. Comparison of model predictions with new measurement data not used in the identification process.

The cross-validation results, as shown in Figure 15, demonstrate how the models respond to new measurement data not used in the identification process. This validation is critical for assessing the generalizability and robustness of both the analytical model and the RBF-based model.

The results from the cross-validation, as depicted in the figure, closely resemble the trends observed in Figure 12. The RBF model exhibits excellent agreement with the experimental data across all tested variables, including position, velocity, and acceleration. The analytical model continues to capture the general trends well, particularly in steady-state behavior, but shows limitations in transient dynamics.

The RBF network model demonstrates excellent generalizability by accurately predicting system behavior for previously unseen data. This robustness validates the model’s applicability to new conditions without requiring extensive reparameterization.

The main intention of our work was not to compare the RBF model with the best possible analytical model but rather to demonstrate a systematic approach for deriving a dynamic model for complex DE systems by combining physics-based approaches with the useful extension of data-driven models in order to better capture inaccuracies and uncertainties with comparatively little effort. In the analytical model a perfectly symmetrical configuration was assumed with identical parameters for the DE actuators. Of course, the model could be modified and refined to match the experimental results more closely. To achieve this, it would be necessary to not only adjust the parameters describing the material behavior of the DE material and the beam but also to account for geometric asymmetries.

The entirety of all parameters could then be adjusted using an optimizer by minimizing the sum of squared errors. Since this problem represents a nonlinear optimization problem with multiple parameters, it is well known that multiple local minima may exist, and there is no guarantee that a global minimum can be reached. Overall, these steps lead to a highly complex and time-consuming process of model improvement and optimization.

To simplify the parameterization of the model for new actuator prototypes in systems and control engineering applications, the RBF model was introduced. The complex analytical modeling process could be replaced by a simple model based on RBF networks, which are easier to parameterize since the unknown parameters enter linearly. Consequently, the resulting optimization problem is a simple convex problem for which a unique global minimum always exists.

While the proposed RBF model offers notable advantages in terms of accuracy and interpretability, it also faces inherent limitations. A central challenge is its limited scalability in high-dimensional or strongly nonlinear input spaces. As the input dimensionality increases or the system exhibits complex nonlinear behavior such as sharp gradients, discontinuities, or abrupt transitions in behavior, the number of required basis functions can grow rapidly, resulting in a significantly enlarged parameter space and substantial computational effort. This phenomenon, often referred to as the curse of dimensionality, may hinder the model’s efficiency and generalizability in more complex scenarios.

Another important aspect is the need for sufficient excitation of all relevant dynamic modes during the identification phase. The model is only capable of capturing physical behaviors that are adequately stimulated in the training data. Consequently, its predictive performance is restricted to the domain spanned by the experimental input signals. Extrapolation beyond this range may lead to significant inaccuracies due to the inherently local approximation properties of RBF networks. However, by carefully designing the excitation signals during experiments, it is possible to ensure that the system operates within the trained domain during subsequent use, thereby avoiding the need for extrapolation.

The risk of overfitting must also be taken into account. A large number of basis functions or excessive overlap (e.g., $\sigma_{\text{n}}\ll 0.5$ ) can introduce strong correlations among parameters, thereby reducing identifiability and compromising model robustness. However, due to the linear-in-parameters structure of the RBF formulation, the regression matrix can be systematically analyzed. By evaluating its rank and condition number, potential overfitting issues can be detected and mitigated.

The performance of the RBF-enhanced model depends on the careful selection of hyperparameters such as the number and width of basis functions. These must be chosen to balance model accuracy with generalization capability.

## 6 Conclusion

This work successfully demonstrates the development and implementation of a hybrid modeling framework for a bistable dielectric elastomer (DE) actuator system, combining physics-based analytical methods with radial basis function (RBF) neural networks. The proposed bistable DE actuator is analyzed in terms of its mechanical configuration and bistable characteristics. A physics-based modeling approach is employed to capture the key features of the actuator, leading to the derivation of a reduced-order model based on analytical considerations. To improve the accuracy and to achieve generalizability of the model, a hybrid approach is introduced, integrating physics-informed structures with data-driven methodologies. The model is enhanced by incorporating experimental data and RBF neural networks.

While the analytical model performs well for design purposes and captures general characteristics, it requires significant parameter tuning for each physical actuator prototype. In contrast, the RBF-based model can easily adapt to uncertainties in assembly, making it more flexible and efficient. The proposed approach effectively addresses challenges posed by nonlinearities and unmodeled dynamics, ensuring a highly accurate representation of the system’s performance. Experimental results confirm the model’s capability to capture both static and dynamic behaviors, including transient responses and bistable switching, with high fidelity. By combining physical principles with data-driven methods, the model achieves improved accuracy and generalizability, while reducing the necessity on extensive datasets. Future research will aim to use the proposed model for the development of a model-based controller specifically tailored to the bistable dielectric elastomer actuator, enhancing its performance and control accuracy.

## Data Availability

The raw data supporting the conclusions of this article will be made available by the authors, without undue reservation.
